# Older Adults With Hearing Loss Have Reductions in Visual, Motor and Attentional Functioning

**DOI:** 10.3389/fnagi.2018.00351

**Published:** 2018-11-06

**Authors:** Susan M. Gillingham, Antonino Vallesi, M. Kathleen Pichora-Fuller, Claude Alain

**Affiliations:** ^1^Baycrest Health Sciences, Rotman Research Institute (RRI), Toronto, ON, Canada; ^2^Department of Psychology, University of Toronto, Toronto, ON, Canada; ^3^Padova Neuroscience Center & Department of Neuroscience, University of Padova, Padova, Italy; ^4^San Camillo Hospital IRCCS, Venice, Italy; ^5^Institute of Medical Sciences, University of Toronto, Toronto, ON, Canada; ^6^Faculty of Music, University of Toronto, Toronto, ON, Canada

**Keywords:** attention, visual reaction time, temporal preparation, variable foreperiod, sequential foreperiod, hearing loss in older adults

## Abstract

Evidence from epidemiological, laboratory and clinical research suggests a link between age-related auditory declines and domain-general cognitive declines. Nevertheless, few studies have experimentally compared measures of non-auditory cognitive functions in younger normal hearing adults (YN), older adults with typical hearing thresholds for their age (ONHA) and older adults with clinically significant threshold hearing loss (OHL). The current study investigated the differences between these groups on measures of attentional response selection and execution to visual stimuli. A visual reaction time (RT) paradigm involving four tasks with differing and hierarchical attentional demands was administered. RTs on trials with differing foreperiods (FPs; pre-stimulus waiting times) were analyzed to assess context-related slowing, error commission and related cognitive control and strategic and automatic neural preparatory processes. The OHL group demonstrated a general slowing that was most apparent on the simplest tasks. Although the number of errors was similar when comparing all three groups, the OHL group exhibited less control over recovery from an error compared to the younger and ONHA groups. Unlike the YN and ONHA groups, the OHL group also showed difficulties with both strategic and automatic response preparation, although automatic preparation was more affected across all tasks. This pattern of results suggests that in older adults with hearing loss there is an underlying difficulty in automatic temporal processing that can affect higher order cognitive functions, although there may not be a completely generalized decline in cognitive functioning that is associated with hearing loss.

## Introduction

Epidemiological, laboratory and clinical studies have provided evidence of an important association between hearing loss and cognitive decline in older adults (e.g., Lindenberger and Baltes, 1994; Baltes and Lindenberger, 1997; Li and Lindenberger, 2002; Lindenberger and Ghisletta, 2009; Humes et al., 2013; Humes and Levi, 2016). The need to understand the mechanisms underlying the association between auditory and cognitive declines is driving ongoing research, especially given the potential for shaping clinical practice with respect to maintaining cognitive health and delaying dementia in older age (for reviews see Martini et al., 2014; Livingston et al., 2017). However, hypotheses concerning the possible mechanisms underlying this association have highlighted the complicated relationship between age-related changes in sensory/perceptual (e.g., audibility, auditory temporal processing), cognitive (e.g., effortful attending and working memory) and socio-emotional (e.g., social isolation) factors (Baltes and Lindenberger, 1997; Tun et al., 2012; Lin et al., 2013; Albers et al., 2015; Pichora-Fuller et al., 2016, 2017). The present study addressed identified gaps in the research that has accumulated over the past 25 years. Specifically, there is a need to further address the issue of sense-specificity by using non-auditory tasks, especially given the observed extra recruitment of non-auditory processing in older adults when presented with auditory stimuli (Ouda et al., 2015). There is also a need to use simpler, attention-based cognitive tasks that may tap basic processes underlying the cognitive abilities elicited by the complicated, working memory-based auditory tasks that have been used in many studies (Grassi and Borella, 2013). As reviewed in the following paragraphs, the research so far has shown that the association between hearing loss and cognitive decline may not be limited to auditory cognition, although it does not necessarily represent a generalized decline in total cognitive functioning. However, there are still limitations to our ability to identify specific cognitive processes that may be affected due nature of many experimental tasks. This study used an experimental paradigm known to engage simple visual attentional processes hierarchically across multiple tasks so that the functioning of specific aspects of cognitive processing could be tested within the context of aging and hearing loss.

A generalized association between sensory and cognitive decline was predicted from early clinical observations in dementia and correlational research. Although the definition of dementia focuses on the end-stage connection between changes in cognition and daily functional activities, changes in motor and multiple sensory systems (e.g., audition, vision, touch) can sometimes be observed many years before a measurable decline in cognition (reviewed in Albers et al., 2015). A major issue with clinical observations, however, is that there is yet no consistent pattern or timeline to the presentation of these symptoms that can be connected to the subsequent development of dementia (Humes et al., 2013). In more formal empirical study of multiple sensory system involvement, the assessment of visual-based cognition is often employed due to our extensive knowledge of the visual system and available tests. In a longitudinal study, Lin et al. (2013) assessed older adults on short measures of global cognitive functioning and executive-based processing speed using visual stimuli over a 6-year period. They found that the cognitive performance of the older adults with hearing loss (assessed using audiometric pure-tone measurements) worsened progressively faster compared to those with better hearing. It was predicted that they would reach a threshold of change that indicated impairment approximately 3 years faster than older adults without hearing loss. This relationship between hearing loss and cognitive decline was independent from potential confounders such as age or cardiovascular risk factors.

Large-scale studies using modeling approaches and/or epidemiological data have provided mixed results with respect to the relation between hearing loss and multiple stages of visual processing during cognitive testing. An early structural equation modeling approach used to study a large sample of hearing aid users without dementia showed that hearing loss was selectively and negatively correlated with verbal long-term memory performance over and above any influence of visual acuity (Rönnberg et al., 2011). However, a subsequent epidemiological study showed that hearing loss in non-hearing aid users was also related to performance on short-term and long-term visual memory tests, although as with the 2011 study, the association was stronger for long-term memory than for short-term memory (Rönnberg et al., 2014).

Small-scale studies examining visual cognition in the context of measurable hearing loss have provided evidence in support of multi-domain involvement in sensory/cognitive decline. Starting with early-stage hearing loss, Campbell and Sharma (2014) found evidence of cortical plasticity in middle-aged (mean = 50 years) adults by measuring visual evoked responses to simple and passive shape-change detection (i.e., no response required). Compared to middle-aged adults with normal audiometric hearing thresholds, there were changes in visual evoked responses in participants with hearing impairments, which correlated with poorer performance on a separate auditory test of speech-in-noise understanding. The authors proposed that changes in visual processing (i.e., perhaps recruitment of visual areas to compensate for hearing loss, such as by focusing more attention on visual cues like facial movements) further reduce auditory processing by recruiting neural regions away from that sensory domain. Studies of older adults with hearing loss also suggest that there may be declines in the visual system as well as the auditory system. For instance, Guerreiro and Van Gerven (2017) assessed 44 older adults (mean ages between 65 years and 67 years across two studies), 22 with better thresholds and 22 with poorer thresholds as defined by a median split of pure-tone hearing thresholds. The cognitive tasks included auditory and visual working memory tasks (1- and 2-n-back tasks, one in each domain) and a reaction time (RT) task that involved inhibitory control (the Stroop task). Their results showed that older adults with poorer hearing performed worse on these tasks compared to younger adults and older adults with age-normal hearing loss, regardless of the primary sensory modality of the task or the relative level of difficulty of each task. However, the tasks used in these studies were cognitively demanding, requiring high levels of cognitive control, manipulation of information within working memory and sometimes the assessment of long-term memory processes. Thus, it is difficult to generalize about the nature of this association between sensory loss and cognitive decline.

For the current study, we intended to further characterize the effect of hearing loss on potentially domain-general cognitive functions by using visual tasks that recruit additional attentional processes as they change progressively in task demands. We utilized four visual attention tasks that initially required basic attending (simple consistent responding to all stimuli) with subsequent additional attentional requirements with each successive task. The four tasks comprise a visual RT paradigm developed by Stuss and colleagues called the Feature Integration Task (FIT; Stuss et al., 1989a,b, 2002; Hetherington et al., 1996).

The paradigm was developed upon the hypothesis that a RT collected at the moment of the response represents the sum of the timing of the stages involved for that particular response to have occurred (Sternberg, 1969). When additional complexity is added to subsequent tasks, the difference in RT between each task represents the extra time taken for the additional cognitive processes required to deal with the task demand (Sternberg, 1969; Stuss et al., 2002). A key component of the FIT task paradigm as designed by Stuss and colleagues is that, in addition to the step-wise introduction of complexity, the integration of features reflecting this complexity is achieved using a single stimulus presented in a center of the screen. The use of a single stimulus allows for reassurance that extra processing at each step is attributed to the processing of just that task-relevant stimulus, and not to extra activities such as scanning a larger search space.

The research findings by Stuss and colleagues using this series of tasks identified: (i) the negative effect of head injury on general processing speed and consistency, divided attention (Complex RT task) and focused attention (Redundant RT task; Stuss et al., 1989a,b); (ii) the negative effect of aging on general processing speed with choice decisions and the disruption of focusing attention in the presence of irrelevant information (Stuss et al., 1989b); (iii) the additional negative interaction of aging and head injury on processing speed and the continuation of improvement of performance consistency more than 5 years after injury (Hetherington et al., 1996); and (iv) the differential negative effect of frontal focal lesion location on general processing speed (maintaining intention to attend, superior medial frontal lobes), error production to specific types of stimuli (false positives (FPs) relating to setting task rules, controlled by the left frontal lobe, and all error types relating to monitoring performance against those rules, controlled by the right frontal lobe) and inconsistency of performance (Stuss et al., 2002, 2003).

The current study employed the measurement of the foreperiod (FP) effect in addition to the measurements (RT, number of errors, and the RT on trials surrounding an error trial) that have been examined previously using the FIT task paradigm. The FP as defined by the tasks used in the current study is the time interval between the response to the previous stimulus and the onset of the next stimulus. Analysis of RT as a function of the length of the FP was included because this measurement provides insight into the internal pre-response preparations that underly a person’s readiness to attend and develop an optimal processing state before responding once the imperative stimulus appears. Evidence suggests that a decline in these preparatory processes contributes to age-related slowing (Kolev et al., 2006). Further study of the FP using both simple and easy binary choice RT tasks similar to those used in this study has shown that preparation to attend and respond may possibly differ in nature. One outcome measure of response preparation, called the variable FP (v-FP) effect, refers to faster response speed as the length of the FP increases (Woodrow, 1914; Karlin, 1959; Drazin, 1961; and reviewed in Niemi and Näätänen, 1981). However, the RT on a given trial is also influenced by the FP that occurred in the preceding trial, labeled the sequential FP (s-FP) effect (Woodrow, 1914; Karlin, 1959), which presents as an asymmetrical distribution of RT dependent upon the trial-to-trial combination of relative FP lengths. In healthy adults, the asymmetry is characterized by slower RT on trials where the FP of the preceding trial is longer than the current trial, in comparison to trials where the preceding FP is either the same or shorter than the current FP.

Regardless of the type of task used, the typical interpretation is that the v-FP effect is thought to result from a strategic evaluation of the passing of time throughout the FP such that the longer the FP, the more likely (i.e., the higher the conditional probability) that the stimulus will appear and thus the more ready the participant is to respond to the incoming stimulus (Näätänen, 1970). Stuss et al. (2005) showed that the benefit associated with increasing the FP is markedly reduced in patients with right frontal lesions, suggesting that the prefrontal cortex plays an important role in strategic time monitoring. However, this conditional probability account does not explain asymmetric s-FP effects and the equal benefit of a preceding short FP when the current trial’s FP is also of an equally short duration. Several theories have been proposed to explain the association between the v-FP and s-FP effects. Los and colleagues (Los and van den Heuvel, 2001; Los et al., 2001) proposed a single process account, which assumes that there is an inherently dependent relationship between the two effects due to the automatic nature of temporal estimation. Alternative accounts suggest that the v-FP and s-FP effects represent independent and separable automatic vs. strategic preparation processes based on lesion and developmental and experimental psychology studies (Stuss et al., 2005; Vallesi et al., 2007, 2014a; Vallesi and Shallice, 2007).

In the current study, RT, error commission and error-related RT, and the v-FP and s-FP effects were used to assess for the functioning of attentional processes in response to differing task demands in the visual modality in younger adults with normal hearing (YN), older adults with normal hearing for their age (ONHA), and older adults with mild hearing loss (OHL). In particular, RT was examined as a function of the FP effect to assess if there were differential changes in underlying automatic vs. strategic preparatory temporal processes in relation to hearing loss that may be above and beyond normal aging. We hypothesized that there may be alterations in sensitive measures of visual attention and response readiness as a function of hearing ability which would further corroborate the links between hearing loss and general cognitive functioning.

## Materials and Methods

### Participants

There were three groups of participants: 21 healthy younger adults (YN; eight males; age: 20–30 years, *M* = 23.3, *SD* = 3.3; education: *M* = 16.8 years, *SD* = 2.1), 17 older adults with typical hearing thresholds for their age (ONHA; three males; age: 66–77 years, *M* = 71.0, *SD* = 3.2; education: *M* = 15.9 years, *SD* = 3.3), and 15 older adults with mild hearing threshold elevations (OHL: eight males; age: 65–79 years, *M* = 73.0, *SD* = 3.8; education: *M* = 16.3 years, *SD* = 3.3). Handedness was determined by asking participants to identify the hand that they used for common tasks such as writing and using a spoon. All participants were community-dwelling and recruited from the participant database at Baycrest Health Sciences, Toronto. Inclusion criteria based on self-report included good English fluency, no history of brain injury and no history of environmental exposure to noise. The study protocol was approved by the Research Ethics Board of Baycrest Health Sciences and carried out in accordance with their recommendations. Informed written consent was obtained according from all participants in accordance with the Declaration of Helsinki.

### Measures

Hearing thresholds were measured and used to categorize the older participants into the ONHA and OLH groups. Other sensory-motor and cognitive measures were administered to describe characteristics of the participants that might influence their performance on the experimental tasks.

#### Hearing

**Figure 1:**
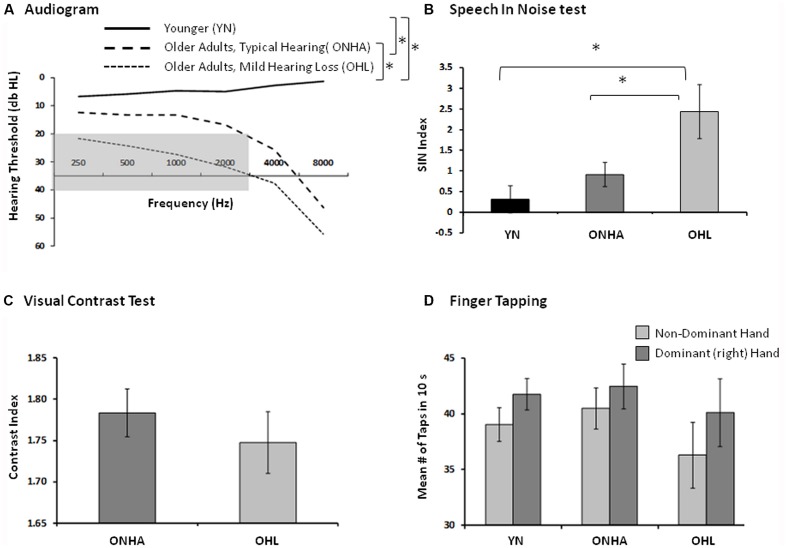
Indices of sensory and motor functioning. The asterisk (*) represents between group differences in performance. **(A)** The group pure-tone averages (both ears). The gray area represents the criteria used to categorize mild hearing loss, audiometric thresholds falling between 20 db and 40 db HL from 250 Hz to 3,000 Hz. **(B)** The Speech in Noise (Quick SIN) index of the amount of target information (words in a sentence) that could be correctly recognized in the context of an increasing amount of background noise (babble). A higher score represents worse performance. **(C)** The index of visual contrast, the number of letters identified (accounting for errors) as the contrast between the target letters and the background gradually decreases. A higher score represents better functioning. **(D)** The index of basic motor speed using the finger tapping test, in both hands, the average number of taps produced in 10 s. A higher score represents better functioning.

The criteria for categorizing the older participants into the ONHA and OHL groups were based on audiometric hearing thresholds. Those in the OHL group had pure-tone air-conduction thresholds obtained in a sound-attenuating booth using standard audiometric procedures that were in the range from 20 dB to 40 dB hearing level (dB HL) for five test frequencies (250, 500, 1,000, 2,000, 3,000 Hz) in each ear. Those in the ONHA group had thresholds below 20 dB HL at each of the five test frequencies in each ear, but some had higher thresholds at 4,000 or 8,000 Hz. For all participants, the pure-tone threshold difference between each ear did not exceed 15 dB at any frequency. The audiometric results averaged for each group of participants are presented in Figure 1A.

#### Other Sensori-Motor Measures

The sensory-motor measures included a speech-in-noise test and a finger tapping test. Visual contrast sensitivity was also tested in older participants. The speech-in-noise test was the Speech in Noise (Quick SIN; Killion et al., 2004) whereby listeners repeated as much as possible of a series of sentences presented in increasing background crowd noise presented via ear inserts (ER-3; Etymotic Research). Six sentences were presented at 65 dB HL in each of two equivalent conditions (using different sentences) in which the signal-to-noise ratio (SNR) decreased in 5 dB intervals from 25 dB to 0 dB SNR with each successive sentence. A SNR index accounted for the average number of critical words repeated across both conditions, with a lower SNR index representing better performance. The QuickSIN results averaged for each group of participants are presented in Figure 1B. Visual acuity of the older participants was assessed via the Mars Letter Contrast Sensitivity Test (Mars Perceptric Corp, 2003–2014) and results are presented in Figure 1C, but it was not tested in younger participants. Given the hand-motor response (button press using two fingers of the dominant hand) required in the experimental task, basic finger tapping speed was assessed in each via the finger tapping test (Reitan and Wolfson, 1993), and results are presented in Figure 1D.

#### Neuropsychological Tests

The main experimental task was expected to utilize multiple cognitive processes including processing speed, simple attention and working memory. Thus a comprehensive but brief neuropsychological battery was administered to all participants in order to provide a standardized measurement of these processes in a similar manner as had been done in previous research (Lin et al., 2013). The tests included the Trail Making Test (parts A and B; Reitan and Wolfson, 1985), Digit Symbol Coding (Wechsler, 1997a), Digit Span Forward and Backward (Wechsler, 1997b) and Spatial Span Forward and Backward (Wechsler, 1997b). The Trail Making Test part A is a measure of focused attention and speed using visual language-based stimuli (digits), and part B has an additional executive component due to having to continually switch between concepts (i.e., alternating between numbers and letters while putting each string in sequence). Digit Symbol Coding is a measure of speeded and focused attention using visual stimuli only. The forward subscales of both the Digit and Spatial Span tests measure auditory and spatial simple attention, respectively. The backward subscales of each test measure auditory and visual working memory.

#### Experimental Task

**Figure 2:**
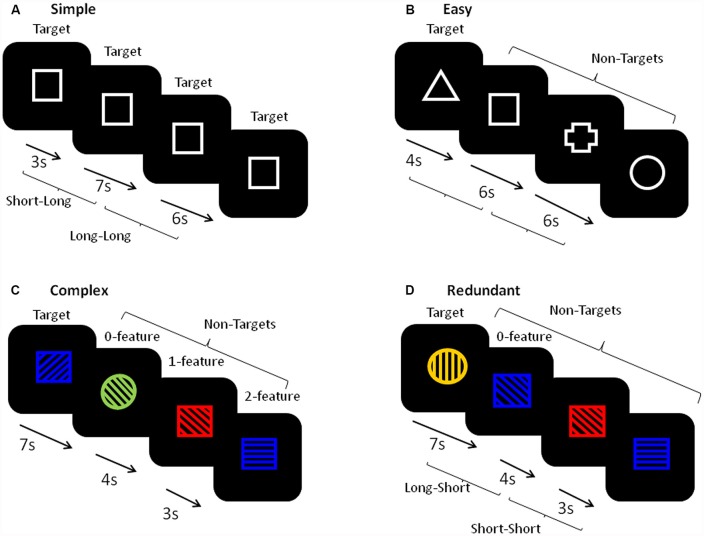
A schematic of the stimuli in each task. **(A)** The simple reaction time (RT) task used a single stimulus and required a single button press in response to each stimulus. **(B)** The Easy task was a binary choice task requiring one button press to the appearance of a pre-defined Target (defined as a white outlined shape, 25%), and a second button press to all other non-target (NT) stimuli (defined as three different white outlined shapes). **(C)** The Complex task was a binary choice task requiring one button press to the appearance of a pre-defined Target (defined as a combination of a shape, color, and directional line filling, 25%), and a second button press to all other NT stimuli (defined by combinations of three features that share either 0, 1, or 2 features with the target). **(D)** The Redundant task was a binary choice task requiring one button press to the appearance of a pre-defined Target (defined as a combination of a shape, color, and directional line filling, 25%), and a second button press to all other NT stimuli (defined by combinations of three features that were not shared with the target). The inter-trial intervals were either 3, 4, 6, or 7 s. For the foreperiod (FP) analyses, trials with both 3 and 4 s FPs were combined to create the ‘short’ FP category and trials with both 6 and 7 s FPs were combined to create the ‘long’ FP category. YN, younger group; ONHA, older adults with typical hearing; OHL, older adults with mild hearing loss.

Figure 2 shows a schematic of the stimuli and presentation procedure. For each task, every trial had a single stimulus appearing in the center of a black computer screen and required a motor hand response with the dominant (right) hand only. There were four different tasks: Simple, Easy, Complex and Redundant. In the Simple RT tasks, participants pressed a button with their index finger in response to the appearance of every stimulus. In the Easy, Complex and Redundant tasks, participants pressed one button with their index finger in response to the infrequent target (25%) and a second button with their middle finger in response to the non-targets (NTs; 75%). Each task block began with a set of either five (Simple RT) or ten (Easy RT, Complex and Redundant) practice trials and on-screen instructions. Following the practice trials, each block contained trials with randomized FPs of four equally frequent lengths (3, 4, 6 or 7 s). There were 50 trials in each block of the Simple RT task and 100 trials in each block of the Easy, Complex, and Redundant tasks. The stimuli in the Simple RT task (Figure 2A) consisted of a simple white-outlined square. Two repetitions of this task were analyzed, one occurring at the beginning and one at the end of the full FIT task battery. The Easy RT task (Figure 2B), occurred immediately after the first Simple RT task. It used four white outlined shapes (square, cross, circle, triangle), one of which was randomly chosen by the software program as the Target for each participant. The Complex task (Figure 2C) used stimuli composed one of the four same shapes as had been used in the Easy task, one of four colors (red, blue, yellow, green), and line fillings that were oriented in one of four possible directions (horizontal, vertical, slanting forward, slanting backward). The target stimulus was defined by a particular combination of the three features, with NTs sharing either 0, 1, or 2 features with the target. The Redundant task (Figure 2D) used stimuli that were defined by three features as in the Complex task, but neither of the NTs shared any of the target’s features.

### Procedure

All participants were administered the sensory-motor tests and the neuropsychological battery and then the experimental tasks. The instructions for the experimental tasks were presented visually on a computer screen at the beginning of each task condition and the experimenter reviewed the instructions with the participant. The target stimulus was randomly generated by the software (E-prime) and the participants were given either 5 (Simple) or 10 (Easy, Complex, Redundant) practice trials for each task condition until they reached the threshold of 80% accuracy on the practice trials required before they proceeded to the experimental task. Responses were collected via a Psychology Software Tools serial response box positioned comfortably under their right hand.

### Outcome Variables

The first outcome variable analyzed was the average RT. Average RTs were calculated across all trial types in the Simple and Easy tasks and for each trial type (target vs. NT) for the Easy, Complex and Redundant tasks. The second outcome variable was the number of errors. The number of errors were tabulated per stimulus type (target and NT) in the Easy, Complex and Redundant tasks. The third outcome variable was the RT of the correct trials both preceding and following an errors trial in the Easy, Complex and Redundant tasks.

Finally, RTs were additionally analyzed according to four categories of trial-to-trial sequential combinations of FP lengths: (i) current short and previous short lengths (short-short); (ii) current short and previous long lengths (short-long); (iii) current long and previous short length (long-short); and (iv) current long and previous long length (long-long). “Short” was defined as FPs of either 3 or 4 s, and “long” was defined as FPs of either 6 or 7 s. The FP analyses were carried out for only the Simple, Easy and Redundant tasks. The RT data from the Complex task was not included in this analysis because it was thought that the higher order cognitive processing required for this task could interfere with the detection of automatic and strategic preparatory activity.

### Data Analysis

All analyses were carried out initially using the sensory (visual contrast) and motor functioning (finger tapping) measurements as covariates. Since the resulting pattern did not change when covariates were included, the results without the covariates are presented.

Scores on the neuropsychological tests were analyzed separately in one-way ANOVAs to evaluate differences between groups.

All RT variables were first analyzed across all tasks in an omnibus repeated measures ANOVA. For the RT and number of errors, they were first compared across the two repetitions of the Simple tasks in a 2 (task repetition) × 3 (group) mixed ANOVA.

Dependent upon the presence of interaction effects, RTs were analyzed in several sequential stages as described below to evaluate if they were affected by the hypothesized attentional demands introduced for each task. RTs were compared between the first Simple task and the overall Easy task (all trial types included) in a 2 (task) × 3 (group) mixed ANOVA to assess for the effect of the introduction of a basic choice on RT. On the choice-based Easy, Complex and Redundant tasks, RTs were first assessed in a 3 (task) × 2 (trial type, target and NT) by 3 (group) mixed ANOVA to assess for the effect of context complexity on RT when confronted with choice. For the Complex task, only the 0-feature NT and the target stimuli were included so that there would be two types of trials as in the other two tasks. The error-related RT was analyzed in a 3 (task) × 3 (trial type) × 3 (group) mixed ANOVA.

The FPs were analyzed across combinations of tasks (the two repetitions of the Simple task, the first Simple task and the Easy task, and the Easy and Redundant tasks) in 2 (task) × 2 (current FP, short vs. long) × 2 (previous FP, short vs. long) × 3 (group) ANOVA. The comparison of the Simple RT tasks was used to assess changes in response preparation as a function of time on task and possible fatigue (tonic arousal). The comparison of the Simple tasks to the Easy task was used to assess changes in response preparation as a function of a slight increase in attentional requirements. The comparison of the Easy and Redundant tasks was used to assess changes in response preparation as a function of the presence of more stimulus information in the Redundant task (shape, color, line filling) compared to the Easy task (shape only). Bonferroni corrections were applied to pairwise comparisons.

## Results

### Neuropsychological Results

There were no significant differences between groups on the Digit Span Forward or Backward tests. Younger adults performed significantly better than both older groups on the Trail Making Test A (faster RT, *F*_(2,50)_ = 5.839, *p* = 0.005, $\eta_{\text{p}}^{2}$ = 0.189), Trail Making Test B (faster RT, *F*_(2,48)_ = 4.957, *p* = 0.011, $\eta_{\text{p}}^{2}$ = 0.171), Digit Symbol Coding (greater number of matched digits-to-symbols, *F*_(2,50)_ = 40.319, *p* < 0.001, $\eta_{\text{p}}^{2}$ = 0.617), Spatial Span Forward (larger span, *F*_(2,50)_ = 6.321, *p* = 0.004, $\eta_{\text{p}}^{2}$ = 0.202) and Spatial Span Backward (larger span, *F*_(2,50)_ = 13.144, *p* < 0.001, $\eta_{\text{p}}^{2}$ = 0.345). The two older groups did not differ significantly from one another.

### RT Results

**Figure 3:**
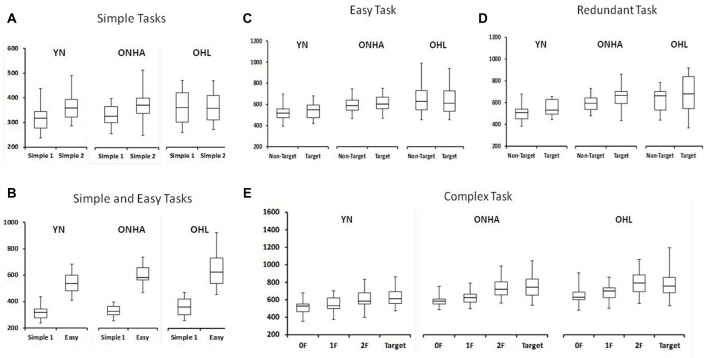
Box plot diagrams showing the RTs for: **(A)** both the Simple RT tasks (the first application of the task is labeled as Simple 1 and the second as Simple 2), **(B)** the first Simple task and the Easy task, and then separated by stimulus type for the **(C)** Easy, **(D)** Redundant and **(E)** Complex tasks. NT, Non-Target. 0F, 1F and 2F refer to the NT stimuli in the Complex task that share either 0, 1, or 2 features, respectively, with the target. YN, younger group; ONHA, older adults with typical hearing; OHL, older adults with mild hearing loss.

Figure 3 shows the RT for each task. There is a significant interaction of group and task (*F*_(8,184)_ = 6.852, *p* < 0.001, $\eta_{\text{p}}^{2}$ = 0.230). This interaction was further examined in the separate ANOVAs examing the tasks in more detail.

#### Simple Tasks

RT in the Simple task condition was slower for the second compared to the first test administration (*F*_(1,49)_ = 25.696, *p* < 0.001, $\eta_{\text{p}}^{2}$ = 0.344). However, a significant interaction between group and task administration highlights that this slowing did not occur for similarly all three groups (*F*_(2,49)_ = 4.030, *p* = 0.024, $\eta_{\text{p}}^{2}$ = 0.141). A one-way ANOVA comparing the change score (the second minus the first administration) illustrated that the YN group had a larger difference between the two administrations compared to the OHL group (pairwise, *p* = 0.028). The ONHA group’s difference score did not differ from either group but was closer to the YN group’s mean. The second administration of the Simple RT task was approximately 50 ms slower than the first for the YN and ONHA groups, whereas it was only 6 ms slower for the OHL group.

#### Simple vs. Easy Tasks

Introducing a choice into the task slowed RTs for all groups, as shown by a main effect of task in the comparison of the first Simple RT task and the Easy RT task (all target and NT stimuli included, *F*_(1,48)_ = 637.632, *p* < 0.001, $\eta_{\text{p}}^{2}$ = 0.930). However, the pattern of RT slowing from the first administration of the Simple task to the Easy task was not the same for all groups (task × group interaction: *F*_(2,48)_ = 5.556, *p* = 0.007, $\eta_{\text{p}}^{2}$ = 0.188). Pairwise comparisons in the ANOVA of the change scores to examine the interaction (Easy task, all stimuli included, minus the first administration of Simple task (*F*_(2,50)_ = 5.556, *p* = 0.007) revealed that the YN group demonstrated less slowing from Simple to Easy compared to both the ONHA (pairwise, *p* = 0.053) and the OHL (pairwise, *p* = 0.008) groups. The two older groups did not differ significantly (pairwise, *p* = 0.738).

#### Effects of Choice and Task Context

The ANOVA comparing the responses to target and NT trial types across the three choice-based tasks (Easy, Complex, Redundant) revealed a main effect of group (*F*_(2,47)_ = 6.839, *p* = 0.002, $\eta_{\text{p}}^{2}$ = 0.225), with younger adults being faster than ONHA (pairwise, *p* = 0.036) and OHL (pairwise, *p* = 0.003) older adults, who did not differ significantly from each other. The main effect of task was also significant (*F*_(2,94)_ = 26.279, *p* < 0.001, $\eta_{\text{p}}^{2}$ = 0.359). Overall, participants had slower RTs on the Complex task than on the Easy (pairwise, *p* < 0.001) and Redundant tasks (pairwise, *p* < 0.001). A main effect of trial type (*F*_(1,47)_ = 56.846, *p* < 0.001, $\eta_{\text{p}}^{2}$ = 0.547) revealed slower RTs to the target than the NT trials.

#### Summary of Behavioral RT Data

The OHL group exhibited a general slowing that was apparent on all tasks, especially on the Simple RT task where, unlike the YN and OHL groups, they demonstrated a slowing on the first administration and then an absence of change from the first to the second administration of the task. Adding a choice component to the response criteria using basic shapes as stimuli (comparing Simple and Easy RT) elicited slowing that was greater for the OHL group compared to the YN group (the ONHA group had an intermediate RT). In the comparison of all three choice-based tasks, there was a general slowing experienced by both older groups in comparison to the YN group, and all groups responded more slowly to the target than to the NT stimulus, regardless of task context.

### Errors

**Figure 4:**
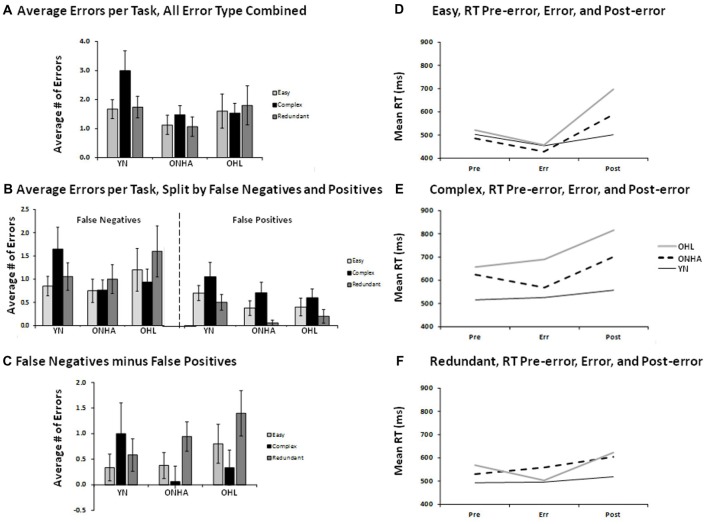
Illustrations for the average number of errors (panels **A—C**) and the RT on error trials and on correct trials preceding and following the error trials (panels **D–F**). Panel **(A)** shows the average number of errors of all types combined for each task. Panel **(B)** shows the average number of errors for each task, split by FNs and FPs. Panel **(C)** shows the difference between the two types of errors (FN minus FPs) for each task. Panels **(D)** to **(E)** show the RT for the trial preceding an error, the error trial, and the trial following an error for each task. FP, false positive; FN, false negative; YN, younger group; ONHA, older adults with typical hearing; OHL, older adults with mild hearing loss.

Overall, few errors were made. The total number of errors did not significantly differ between the three groups, and are depicted in Figures 4A–C. There was a larger number of false negative (FN) compared to FP errors (responding to an infrequent target as though it were a NT) for all three groups, as shown in the main effect of error type (*F*_(1,47)_ = 14.988, *p* < 0.001, $\eta_{\text{p}}^{2}$ = 0.242).

Error-related RT was examined by evaluating the RT on error trials and on the trials immediately preceding and following the error trials, and is depicted in Figures 4D–F. An omnibus ANOVA comparing RT across these three trial types across all three tasks indicated that the groups differed in their RT pattern as a function of task (task × group interaction: *F*_(4,46)_ = 3.637, *p* = 0.012, $\eta_{\text{p}}^{2}$ = 0.240) and by trial type (trial type × group interaction: *F*_(4,46)_ = 3.511, *p* = 0.014, $\eta_{\text{p}}^{2}$ = 0.234). Follow-up analyses examined group differences in each task separately. In the Easy task, a main effect of trial type (*F*_(2,60)_ = 22.730, *p* < 0.001, $\eta_{\text{p}}^{2}$ = 0.431) showed that the post-error trial had a slower RT compared to both the pre-error and error trials (pairwise, *p* < 0.001). A trial type × group interaction (*F*_(4,60)_ = 4.625, *p* = 0.003, $\eta_{\text{p}}^{2}$ = 0.236) was further investigated by performing separate ANOVAs on change scores calculated for the pre-error RT (pre-error RT — error RT) and post-error RT (error RT — post-error RT). There were no group differences on the pre-error RT calculation. As shown in Figure 4D, a group × RT change interaction for the post-error RT calculation (*F*_(2,30)_ = 7.513, *p* = 0.002) illustrated that the YN group’s post-error change in RT was significantly smaller than that of the OHL group (pairwise, *p* = 0.002). The ONHA group also showed slowing on the post-error trial, but the RT cost was of an intermediate value that did not significantly differ when compared to the YN and OHL groups. In the Complex task, again a main effect of trial type (*F*_(2,72)_ = 6.501, *p* = 0.003, $\eta_{\text{p}}^{2}$ = 0.153) showed that the post-error trial RT was slower than both the pre-error (pairwise, *p* < 0.001) and error (pairwise, *p* = 0.021) trials. The trial type × group interaction was not significant as there was post-error slowing for all three groups. However, the main effect of group was significant (*F*_(2,36)_ = 8.756, *p* = 0.001, $\eta_{\text{p}}^{2}$ = 0.327). As illustrated in Figure 4E, the OHL group had an overall RT across all trials that significantly differed from the YN (pairwise, *p* = 0.001). The ONHA group had an intermediate RT that did not significantly differ from either group. For the Redundant task, the difference between trial types was less pronounced for all three groups (Figure 4F), and the post-error RT was only slightly higher than the pre-error trial RT (the difference was not statistically significant).

#### Summary of Errors

There were more FN errors than FPs for all three groups, and this difference between error types was slightly larger in the Redundant task compared to the Easy task. With respect to RTs related to errors, the post-error trials elicited a slower RT compared to the pre-error and error trials for all groups for almost all tasks. In the Easy task, the OHL group showed greater post-error slowing compared to the YN, while the ONHA group had an intermediate amount of post-error slowing. The Complex task elicited post-error slowing in all three groups, although the OHL group was slower across all three trial types compared to the YN and the ONHA group showed an intermediate RT. There was a weak indication of post-error slowing for all three groups in the Redundant task, but there were no significant group differences.

### Foreperiod Effects

**Figure 5:**
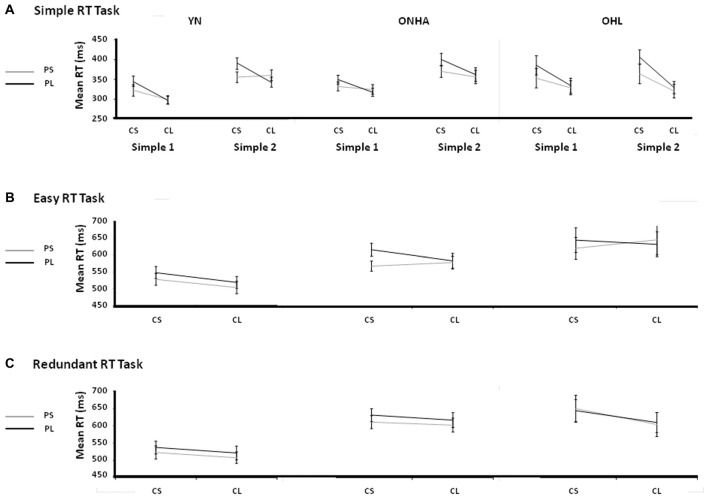
Illustrations of the v-FP and s-FP effects for the Simple **(A)** Easy **(B)** and Redundant **(C)** tasks. CS, Current Short FP; CL, Current Long FP; PS, Previous Short FP; PL, Previous Long FP; Simple 1, the first administration of the Simple task; Simple 2, the second administration of the Simple task; YN, younger group; ONHA, older adults with typical hearing; OHL, older adults with mild hearing loss.

Figure 5 shows the group mean RTs in the various FPs for each task. An initial omnibus ANOVA involving four tasks (first and second administrations of the Simple task, Easy task, Redundant task) revealed the presence of the expected FP effects. RT was faster when the current FP was relatively longer, showing the v-FP effect (main effect of the current FP: *F*_(1,46)_ = 45.684, *p* < 0.001, $\eta_{\text{p}}^{2}$ = 0.498). RT was also faster when the previous trial’s FP was relatively shorter than the current trial’s FP, showing the s-FP effect (main effect of the previous FP: *F*_(1,46)_ = 58.511, *p* < 0.001, $\eta_{\text{p}}^{2}$ = 560). The s-FP effect was modulated according to the length of the previous FP, where relatively longer previous FP’s produced a slower RT, showing the asymmetrical nature of the s-FP effect (current × previous FP interaction: *F*_(1,46)_ = 24.239, *p* < 0.001, $\eta_{\text{p}}^{2}$ = 0.345). Interaction effects between the FP, task and group variables for the current FP (task × FP × group: *F*_(6,138)_ = 3.119, *p* = 0.007, $\eta_{\text{p}}^{2}$ = 0.119), previous FP (task × FP × group: *F*_(6,138)_ = 2.166, *p* = 0.05, $\eta_{\text{p}}^{2}$ = 0.086) and asymmetrical effects (task × current FP × previous FP: *F*_(3,138)_ = 3.679, *p* = 0.014, $\eta_{\text{p}}^{2}$ = 0.074) showed modulation of the classic FP effects as a function of group membership and task context.

#### Simple Tasks

Comparing the two administrations of the Simple RT task (Figure 5A), the main effect of the current FP was significant (*F*_(1,49)_ = 57.548, *p* < 0.001, $\eta_{\text{p}}^{2}$ = 0.540), showing the classic v-FP effect of a faster RT on trials that have a longer current FP. The main effect of the previous FP was also significant (*F*_(1,49)_ = 33.146, *p* < 0.001, $\eta_{\text{p}}^{2}$ = 0.403), showing the basic s-FP effect of faster RTs when a previous trial had a short FP. The current × previous FP interaction was also significant (*F*_(1,49)_ = 23.304, *p* < 0.001, $\eta_{\text{p}}^{2}$ = 0.322), illustrating the asymmetric aspect (longer RT when the previous trial’s FP is longer than the current trial’s FP) of the s-FP effects. Unlike the YN and ONHA groups, the OHL group had a slower RT on trials that had a short current FP whereas their RT on trials that had a long current FP was almost exactly the same across both tasks (i.e., there was more of a cost to RT when the current FP was short, although the interaction was non-significant, *F*_(2,49)_ = 2.914, *p* = 0.064, $\eta_{\text{p}}^{2}$ = 0.106).

#### Effects of Choice and Task Context

In the comparison of the Simple and Easy tasks, there were again the expected main effects representing the classic v-FP (main effect of current FP: *F*_(1,48)_ = 24.984, *p* < 0.001, $\eta_{\text{p}}^{2}$ = 0.342), s-FP (main effect of previous FP: *F*_(1,48)_ = 29.250, *p* < 0.001, $\eta_{\text{p}}^{2}$ = 0.379), and asymmetry of the s-FP (current × previous FP interaction: *F*_(1,48)_ = 23.146, *p* < 0.001, $\eta_{\text{p}}^{2}$ = 0.325) performance patterns were observed.

A task × current FP interaction (*F*_(1,48)_ = 7.111, *p* = 0.01, $\eta_{\text{p}}^{2}$ = 0.129) illustrated that the difference between the current short and long FPs was smaller in the Easy task (longer FP faster by 10.6 ms) than in the Simple task (longer FP faster by 31.3 ms) when all three groups were considered together. The task × previous FP × group interaction was significant (*F*_(2,48)_ = 4.289, *p* = 0.019, $\eta_{\text{p}}^{2}$ = 0.152). As illustrated in Figures 5A,B, the OHL group did not benefit from having a relatively short FP on the previous trial compared to the current trial in the Easy task as it did in the first Simple task, unlike the YN and ONHA groups.

#### Effects of Interfering Information in the Task Context

In the comparison of the Easy and Redundant tasks, again the expected main effects were present, showing the classic v-FP pattern (main effect of current FP: *F*_(1,47)_ = 15.792, *p* < 0.001, $\eta_{\text{p}}^{2}$ = 0.251), s-FP pattern (main effect of previous FP: *F*_(1,47)_ = 27.239, *p* < 0.001, $\eta_{\text{p}}^{2}$ = 0.367), and asymmetry of the s-FP (current × previous FP interaction: *F*_(1,47)_ = 7.021, *p* = 0.011, $\eta_{\text{p}}^{2}$ = 0.130). The task × current FP × group interaction was significant (*F*_(2,47)_ = 4.602, *p* = 0.015, $\eta_{\text{p}}^{2}$ = 0.164). It indicated that the OHL group had a different response pattern for the short vs. long current FPs across the tasks (Figures 5B,C). On average, the trials with a current long FP were faster than trials with a current short FP (the typical v-FP effect) in both tasks for the YN (Easy, long = 511 ms, short = 538 ms; Redundant, long = 514 ms, short = 530 ms) and ONHA (Easy, long = 589 ms, short = 600 ms; Redundant, long = 603 ms, short = 619 ms) groups. By contrast, for the OHL group, the RTs on trials with a current long FP were very similar, and actually slower, than on the trials with a current short FP in the Easy task (long = 650 ms, short = 643 ms), but the results followed the same pattern as the other two groups in the Redundant task (long = 617 ms, short = 654 ms). That is, they returned to the typical v-FP effect in the Redundant task. Additionally, a previous FP × group interaction (*F*_(2,47)_ = 4.448, *p* < 0.017, $\eta_{\text{p}}^{2}$ = 0.159) indicated that there was a difference between groups in the RT response that was dependent upon the previous trial’s FP. Regardless of task, RTs on trials with a previous short FP were faster than on trials with a previous long FP (the typical s-FP effect) for the YN (Easy, previous short = 514 ms, previous long = 534 ms; Redundant, previous short = 515 ms, previous long = 529 ms) and ONHA (Easy, previous short = 580 ms, previous long = 610 ms; Redundant, previous short = 603 ms, previous long (619 ms) groups. By contrast, the OHL group showed similar RTs to trials with previous short and long FPs in both tasks (Easy, previous short = 643 ms, previous long = 649 ms; Redundant, previous short = 636 ms, previous long = 636 ms). That is, they did not display a typical s-FP effect in either task.

#### Summary of Foreperiod Effects

In the comparison of the two administrations of the Simple RT task, the classic v-FP and s-FP effects, and the asymmetrical pattern of the s-FP effect were present for all groups. An interaction effect with the group, task and current FP variables that trended toward significance indicated that the OHL group may have experienced a greater cost of RT when the current FP was relatively shorter, especially in the second repetition of the task. Notably, with the introduction of choice (comparison of the first Simple RT task and the Easy task), the OHL group, unlike both the YN and ONHA groups, did not benefit through a quickening of the RT on trials with a relatively shorter previous FP. In the two choice-based tasks (Easy and Redundant), the OHL group again did not benefit through a quickening of RT from having a relatively shorter previous FP, but they were able to regain their vFP effect and respond faster on trials with a current long FP as opposed to no benefit to RT in the Easy task.

## Discussion

The purpose of this study was to compare attentional processing in older adults with mild hearing loss (OHL) against that of older adults with age-normal hearing (ONHA) and younger adults (YN) using visual RT tasks that increased in complexity, and thus in the requirement for attentional resources, both within and across tasks. The use of visual RT tasks allowed for the evaluation of domain-general changes across basic attentional processes that may be associated with aging, and the varying level of complexity allowed for the evaluation of the different levels of attentional processing. Both older groups showed comparable performance, with expected age-related changes when compared to the YN group, on processing speed and attention as indexed by performance on neuropsychological tests. Importantly, using visual detection tasks that varied in attentional demands, we showed deficits in attention and response preparation processes in older adults with mild hearing loss that were not observed in younger or older adults who had better hearing thresholds. The findings for the OHL group indicate that there may be underlying disruptions in some cognitive processes that span multiple sensory and cognitive domains, but do not necessarily represent a generalized decline in global functioning.

### General Slowing and Impairments in Self-Guided Engagement

The first overall observation from the data is that of a general slowing demonstrated by the OHL group compared to both the YN and ONHA groups. However, this group-specific slowing was more noticeable in the Simple RT and Easy RT tasks than on the Complex and Redundant tasks. Furthermore, the OHL group showed an unusual pattern of performance across administrations of the Simple RT task. While the YN and ONHA groups showed the expected increase in RT from the first to the second repetition of the task, the RT of the OHL group was a little slower on the first administration than the RT of the other two groups and their RT did not change from the first to the second administration. The RTs on the Easy task were also somewhat unusual for the OHL group, insofar as there was no differentiation between RTs for the target and NT trials, as though they were reaching a sort of ceiling effect in their response (although there was no significant interaction effect on RTs to the different trial types between groups).

Simple RT has been previously reported to be somewhat resistant to the cognitive effects of aging and general slowing (Welford, 1980; Salthouse, 1985; Stuss et al., 1989b). The unusual findings for the OHL group in the current study could reflect difficulty in the initial strategic or voluntary effort required for all higher-order attentional processes, which Stuss and colleagues defined as energization (Stuss et al., 2002, 2005; reviewed in Stuss and Alexander, 2007). The previous findings reported by Stuss and colleagues in studies of patients with brain lesions involving damage to specific dorso-medial frontal lobe regions have been interpreted as evidence of a disruption in this effort system (Stuss et al., 2002; Stuss and Alexander, 2007; Stuss, 2011). The results observed in the OHL group were more subtle than the findings reported in lesion studies insofar as the unusual effects for the OHL group were limited to tasks that depended on mostly self-driven sustained attention (Simple and Easy RT tasks but not the Complex and Redundant tasks) and could indicate a problem attending when the tasks are somewhat easy and monotonous. Also, the almost equivalent performance between the two administrations of the Simple RT task has never been shown before in any previous publications in patients using this FIT task, or in unpublished data in a group of healthy people of similar and older age than the current sample, or other similar tasks (Stuss et al., 2005). One possibility to consider is age-related slowness in motor output, especially given that it has been shown to affect simple RT (Woods et al., 2015) and the non-significant slowing of the non-dominant hand by the OHL group in this study. However, the participant’s hand-motor responses as measured by single finger tapping in the hand that was used to make the response was equivalent across the three groups, although more variable for the OHL group. Additionally, the respective statistically significant findings on the RT measures remained significant when the finger-tapping scores were used as a covariate. Comparatively, although the OHL group is still generally slower than the YN and ONHA groups on the more difficult Complex and Redundant tasks, they showed more differentiation between trials types on these tasks than in their performance on the easier tasks. On these latter two tasks, their slowest responses occurred to the Target stimulus, although they had a little more difficulty in the most demanding scenario of differentiating between the target and 2-feature NT in the Complex task. In these more difficult tasks, there is more stimulus information available which may seemingly drive the system to respond. The slowing from the first to the second administration of the Simple task for the YN and ONHA groups seems to represent a release from the effortful attending on the tasks of intermediate difficulty. For the OHL group, however, the lack of change in RT from the first to the second administration of the Simple task suggests that there may not have been as big of a change in the amount of effort, or drive, to the system with the introduction of choice and additional stimulus features, and that perhaps the system was already working at the optimal pace.

Another possibility to consider is that this type of pattern in performance is similar to what is occasionally observed in populations with affective disorders, especially depression. Many theories expect cognitive performance by people with depression to decline as attentional requirements increase, due to either reduced cognitive capacity (Hasher and Zacks, 1979; Hasher et al., 1985), or the narrowing of attentional focus. By contrast, some studies have shown improved performance with increasing attentional demand (Krames and McDonald, 1985; Hertel and Rude, 1991), including variations on simple RT tasks (Thomas et al., 1999). However, there is a limit to the improvement as people with depression performed worse than controls when there was a decision or choice (Thomas et al., 1999). One interpretation of the improvement in performance with some increase in attentional demand is the sudden utilization of attentional or executive resources that were previously utilized by thought processes related to the affective disorder while the person performed the easier task, and this implies that there was not just a general slowing with depression. In the current study, such an interpretation of distracted attentional resources would not perfectly apply to these older adults with hearing loss because performance is not fully improving on more difficult tasks, but instead there appears to be more engagement of attention when more stimulus information in involved. However, it does suggest that, in these older adults, there may be some general underlying difficulty with their ability to engage attention on their own or when discrimination is difficult, but still have the attention/executive resources to show more engagement when the opportunity arises.

### Executive Impairments in Response Bias and Control

Their error commissions and subsequent recovery provides some evidence that the OHL group had difficulty on the more demanding tasks. Overall, they did not commit more errors than the other two groups, and it was also observed that error commission did not vary with age. The YN group also showed more errors (in the Complex task) than the older groups. However, the OHL group generally committed more FN errors across most tasks, and they had more error and post-error slowing on the Complex task. This suggests that even though they may have been more engaged with these stimuli that had more information to process in the relatively more difficult tasks, the increase in FN errors can represent a response bias and a difficulty in switching from the more common response (responding that an item was a NT was expected 75% of the time) and they had more difficulty with controlling their recovery from making an errors in such a demanding context as contained within the Complex task.

### Mechanisms of Response Preparation

The nature of the slowing in attentional processes was examined through the analysis of the RTs as a function of FP length. In the Simple RT tasks, for all three groups, the classic effects of v-FP (shorter RTs on trials with longer FPs), s-FP (shorter RTs when the previous trial’s FP was shorter than the current trial), and the asymmetric nature of the s-FP effect (longer RTs when the previous trial’s FP is longer than the current trial’s FP) were present. However, the OHL group experienced slowing when the current trial’s FP was short, whereas they were able to respond nearly as quickly as the other groups on trials with a current long FP, and they maintained this ability across tasks. They were actually a little faster on the current long FP trials in the second administration of the Simple task but, especially in comparison to the YN group, they experienced a greater cost (longer RT) on trials that had a current short but a previous long FP.

According to theories that try to explain differences in the underlying processes of the v-FP and s-FP, when the current trial’s FP is short, automatic processes related to motor arousal determine the relative RT dependence upon the previous trial’s FP. If the previous trial’s FP is short, then there is a facilitation effect whereby phasic arousal is increased and the RT will be approximately the same as the RTs on the current long FP trials. However, if the previous trial has a relatively long FP, then there is a temporal refractory period at the motor level that delays responding, and creates the asymmetric nature of the s-FP effect (Vallesi et al., 2007; Vallesi and Shallice, 2007). Across tasks, the YN and ONHA groups demonstrated a shift of their entire distribution of RTs across all FP combinations, reflecting a general slowing from the first to the second task. The slowing for the OHL group, however, appeared consistent across both administrations of the Simple task. This slowing was reflected in difficulty reacting on trials where there was a relatively short FP, and this was exacerbated when the previous trial had a relatively longer FP. This pattern of difficulties related to the length of the FP suggests that participants with mild hearing loss are able to strategically monitor the passage of time when the current FP is relatively long and they are not necessarily affected by changes in tonic levels of arousal. However, they may experience difficulty with the more automatic neural preparatory activity that is responsible for phasic levels of arousal and motor preparedness.

With the introduction of choice, the OHL group did not benefit from having a previous short FP as did the YN and ONHA groups, which generally maintained a consistent pattern of performance across the different FP combinations in all tasks. The YN group had a reduced asymmetry in their s-FP effect in the Easy task because they did not seem to experience as high of a facilitation effect on short-short FP trial combinations. Additionally, in comparing the two choice tasks (Easy and Redundant), the OHL group again did not benefit from having a previous short FP in the Redundant task, but these participants were able to have faster responses on trials with a current long FP in the Redundant task compared to the Easy task. The YN and ONHA groups demonstrated the typical FP effects that have been previously shown using both simple and easy choice tasks (Vallesi et al., 2014b). The change in the OHL group’s performance across tasks, however, suggests that there was an increase in the attention of these older adults to the stimulus identity and selection that significantly altered preparatory abilities. In the transition from the Simple to the Easy task, the increase in cognitive demand changed their ability to rely on the automatic timing aspects that are thought to underlie the sequential effects, but also caused their strategic timing preparatory abilities to be less effective (the disappearance of the v-FP effect). The Redundant task, however, used stimuli that contained much more identifying information (shape, color, line orientation), and the OHL group again experienced disruptions in their automatic timing abilities but were able to engage their strategic timing abilities to a greater degree as shown in the return of their v-FP effect.

### Source of Response Preparation Deficits in Aging and Sensory Loss

The question of interest would be to account for the source of the difficulty of the automatic preparation processes in the OHL group, and to identify why there may be a reduction in this ability. The previous studies on dissociations of the automatic and strategic processes have looked at either brain injury to specific regions that would identify a distinct process (Stuss et al., 2005; Vallesi et al., 2007) or at the development of these processes at a young age, examining when they actually appear for the first time (Vallesi and Shallice, 2007). The lesion studies have identified cortical areas that are necessary to the typical functioning of these processes. For the OHL group, however, there may not necessarily have to be a disruption in a specific cortical area (and it would be difficult to explain why their left premotor region would be particularly affected). Also, a cortical-based disruption in just pure automatic motor preparation would suggest that the same pattern of findings should have appeared across all tasks, whereas the OHL group here show selective effects based upon attentional demand. In the context of the findings on other tasks in this study, such as apparent ceiling RT effects on easy tasks and the increased engagement on more difficult tasks, and even those findings that show non-significant group differences (slight reductions in visual contrast, slight reductions in non-dominant finger tapping), which can be disrupted at multiple points throughout a distributed motor system (Prigatano and Borgaro, 2003), and the reduction in hearing ability, suggests that there may be common underlying neural preparatory processes that are affected in this group. Auditory-based temporal processing is considered a key aspect to successful cognitive performance (Fogerty et al., 2010), and speech understanding (Vaughan et al., 2008) in aging. This current study suggests that there may be underlying disruptions in temporal processing that can be measurable in non-auditory tasks as well. An early hypothesis of mechanisms that underlying the hearing loss-cognitive decline relationship posited a common cause, such as a general neural degeneration underlying both sensory and cognitive decline in tandem (Lindenberger and Baltes, 1994; Baltes and Lindenberger, 1997; Humes et al., 2013). Rönnberg and colleagues suggested that their findings of differential influence of hearing loss on performance on different types of memory tests (long-term more than short-term) is evidence of a selective (rather than general) mechanism that connects hearing loss (more so than vision loss in their case) to specific types of cognitive decline. The results from this current study may not necessarily support a full common-cause association between cognitive and sensory decline. Similar to the results from the Rönnberg et al. studies, deficits are not completely generalized, at least in this very mild stage of hearing loss in a healthy sample of participants.

If the pattern of results here is meaningfully related to some form of underlying disruption in neuronal functioning, it could be hypothesized that such disruptions involve changes in white matter integrity and cortical volume that has been associated with age-related hearing loss. However, there is still much to be investigated with respect to the age of the person at time of the onset of hearing loss and the health of neuronal tracts outside of the auditory system (reviewed in Mudar and Husain, 2016). Changes in white matter integrity might be expected to have an even greater general effect that would have affected the OHL group’s level of tonic arousal as well. Another possibility is a disruption earlier in the pathway affected overlapping areas for sensory and motor information transfer and integration, such as the thalamus (Cappe et al., 2009).

## Conclusion

The present results indicate that there may be some common disruptions in neural processes that affect multiple systems underlying cognitive changes that have been previously observed to be linked to hearing loss in older adults. Indicators of attention-related differences in the OHL group were observed on the simplest of tasks (generalized slowing, a lack of change in the RT response in the context of changes in effort or a release from effortful attending). Additionally, a combination of increased engagement in the more difficult tasks and the continued disruption of automatic response preparatory abilities while strategic preparatory abilities improved when there was more stimulus information to attend to highlighted possible disruptions in automatic timing processes that underlie higher cognitive functions. These results also suggest that, although there may be involvement of multiple sensory and motor systems as would be expected by the common cause hypothesis, the outcomes on behavior may not be fully generalized as the OHL group still exhibited intact strategic attentional abilities under certain contexts (e.g., intact vFP effect in the Simple and Redundant tasks). In this case, the interaction between the neural disruptions and measurable effects on higher order cognitive functions may depend on variables such as age of onset and time since onset of hearing loss and genetic or environmental factors affecting neuronal health. This study specifically included participants who were cognitively and functionally healthy. A goal of the study was to use a task with a well-established empirical base to evaluate if changes in basic attentional processes are associated with mild hearing loss and detectable before there is any significant functional decline. Therefore, replication is required to test the stability of these findings. Nevertheless, a strength of the study was using multiple measures with different task demands. This allowed us to view a consistency in the small effects using different outcome measures showing that the OHL group was not performing the same as the ONHA group, and that there was a manipulation of the outcome variables across different measures as a function of the changing task demands. In addition to replication, future research should investigate whether the effects seen here are related to the same sensory-cognitive changes noted in even earlier-onset hearing loss (Livingston et al., 2017) or the source of the variability that seems to be a bit higher in the OHL group, and to disentangle the effects of early motor preparation and higher order attentional processing, and their interdependency.

## Author Contributions

SG: study conceptualization, study design, data collection and analysis, interpretation and writing. AV: design of data analysis, interpretation and writing. MP-F and CA: study conceptualization, study design, interpretation and writing.

## Conflict of Interest Statement

The authors declare that the research was conducted in the absence of any commercial or financial relationships that could be construed as a potential conflict of interest. The reviewer MG declared a shared affiliation, with no collaboration, with one of the authors AV, to the handling editor at time of review.
